# hECA v2.0: an AI-ready ensemble cell atlas of single-cell RNA and ATAC sequencing data

**DOI:** 10.1038/s41597-025-06426-2

**Published:** 2025-12-15

**Authors:** Xi Xi, Yixin Chen, Xinze Wu, Minsheng Hao, Jiaqi Li, Haiyang Bian, Qiuchen Meng, Fanhong Li, Chen Li, Chuxi Xiao, Xiaomin Dong, Renke You, Yifan Xiong, Peng Yang, Zijing Gao, Xuejian Cui, Yan Pan, Zhen Li, Wenrui Li, Zhuofeng Li, Xiaoyang Chen, Yanfei Cui, Hairong Lv, Rui Jiang, Lei Wei, Xuegong Zhang

**Affiliations:** 1 MOE Key Laboratory of Bioinformatics and Bioinformatics Division of BNRIST, Department of Automation, Tsinghua University, Beijing, China; 2 Department of Neurology, Aerospace Center Hospital, School of Life Science, Beijing Institute of Technology, Beijing, China; 3 School of Medical Engineering, Affiliated Zhuhai People’s Hospital, Beijing Institute of Technology, Zhuhai, China; 4 Department of Neurology, Zhongshan Hospital and Institute of Science and Technology for Brain-Inspired Intelligence, Fudan University, Shanghai, China; 5 State Key Laboratory of Brain Function and Disorders, Institutes of Brain Science, Fudan University, Shanghai, China; 6 MOE Frontiers Center for Brain Science, Fudan University, Shanghai, China; 7 Department of Psychological and Cognitive Sciences, Tsinghua University, Beijing, China; 8 Fuzhou Institute of Data Technology, Fuzhou, China; 9 Center for Synthetic and Systems Biology, School of Life Sciences and School of Medicine, Tsinghua University, Beijing, China

**Keywords:** Data integration, Data publication and archiving

## Abstract

With the growing accumulation of scattered single-cell data and the rapid advancement of artificial intelligence (AI), there is a pressing need for a high-quality, well-organized, and AI-ready single-cell data resources to support large-scale model. Here, we present version 2.0 of human Ensemble Cell Atlas (hECA), a cell atlas incorporating both single-cell RNA sequencing (scRNA-seq) and single-cell ATAC sequencing (scATAC-seq) data. It expands the scRNA-seq data collection to 10,831,024 human cells with unified labels, and adds the new modality of scATAC-seq profiles with 1,450,511 cells. The data cover 42 human organs and tissues. To ensure cross-dataset consistency and quality, we standardized gene expression and chromatin accessibility matrices, harmonized cellular metadata, and manually re-annotated cell types based on the unified Hierarchical Annotation Framework (uHAF). The strength of the dataset has been shown in pre-training the large generative cellular AI model scMulan. hECA2.0 provides a well-structured and ready-to-use data resource, serving as a robust data foundation for AI-driven single-cell research.

## Background & Summary

The past decade has witnessed a rapid accumulation of single-cell sequencing data from labs across the world. Since the introduction of the first single-cell RNA sequencing (scRNA-seq) technology in 2009^1^, massive amounts of scRNA-seq data have been generated using diverse sequencing platforms across multiple laboratories worldwide. Concurrently, the development of single-cell sequencing technologies targeting other modalities has also progressed rapidly, offering insights in biological processes such as epigenetic regulation and translation^2^.

Large-scale initiatives, such as the Human Cell Atlas (HCA) and the Human BioMolecular Atlas Program (HuBMAP), aim to construct comprehensive atlases of human cells to advance our understanding of health and disease^3–6^. The advancement of these consortia, together with the massive accumulation of single-cell data from numerous independent studies, has collectively driven the development of a wide range of databases and data portals that integrate and standardize scattered single-cell datasets, including those for scRNA-seq.^7–18^, single-cell assay for transposase-accessible chromatin using sequencing (scATAC-seq)^19^, single cell bisulfite sequencing (scBS-seq)^20^, and single-cell multi-omics data^21–23^.

With the recent rise of artificial intelligence (AI) and its growing impact on scientific research, single-cell omics are entering an AI-driven era. A key emerging goal is to develop large cellular models and AI virtual cells (AIVCs)^4,24^. This underscores the growing importance of constructing an AI-ready single-cell omics atlas, which will serve as an important data foundation for AI-driven biological research. There are several key considerations in building such an atlas. First, the atlas should include data collected from a wide range of sources, encompassing different organs, cell types, donor ages, and other relevant biological variables, with accurately and comprehensively recorded metadata to preserve biological contexts. Second, data from different sources should undergo standardized processing to ensure consistency in feature representation and metadata annotation. Third, the resulting atlas should be well-organized, structured, and easy to access to facilitate convenient use.

In our earlier work, we built the human Ensemble Cell Atlas (hECA) v1.0^9^, a cell atlas based on the concept of “cell-centric assembly”. This initial version integrated 1,093,299 scRNA-seq profiles using standardized preprocessing and manual annotation guided by the unified Hierarchical Annotation Framework (uHAF)^25^, improving consistency across studies.

**Fig. 1:**
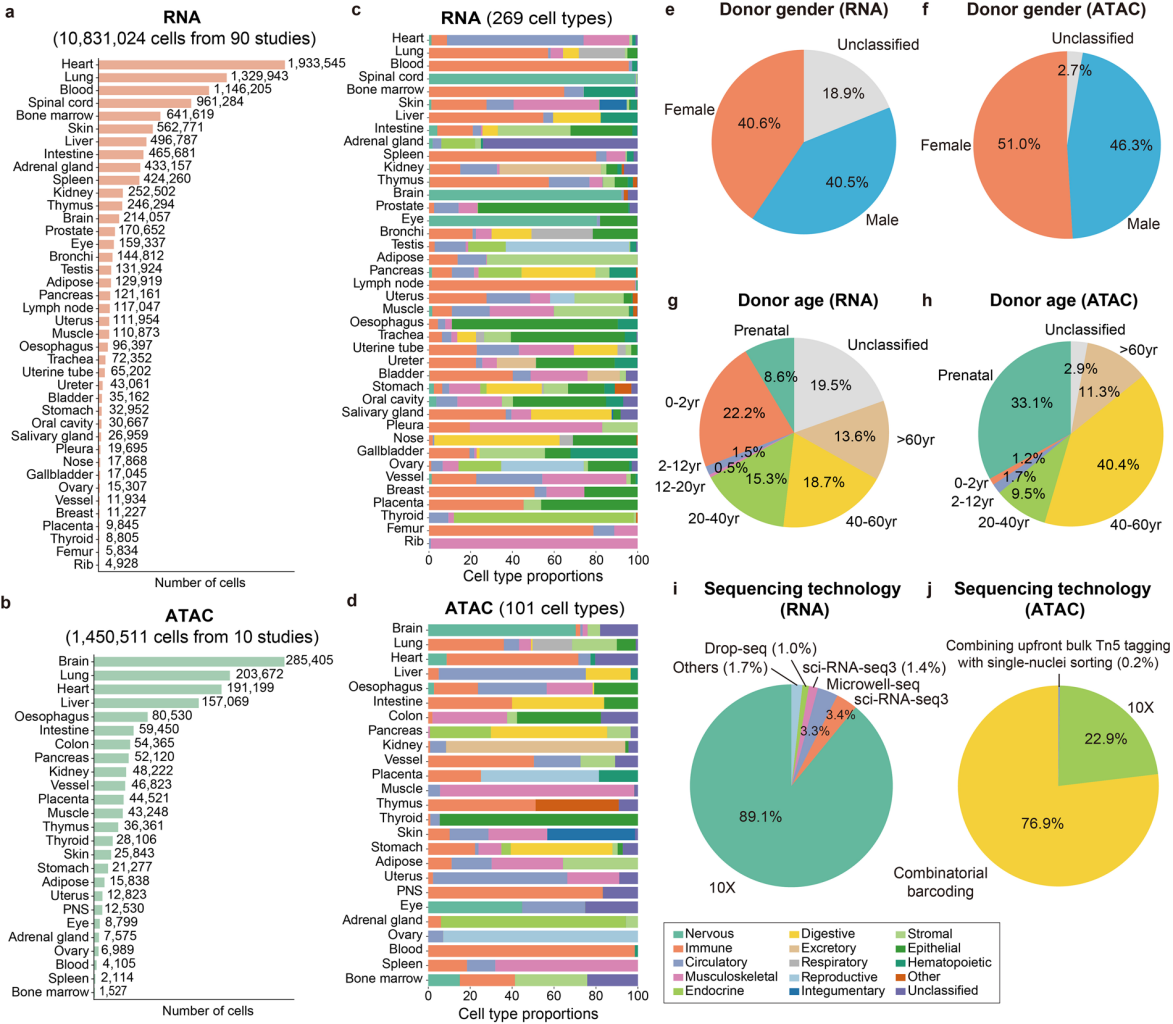
Overview of hECA v2.0. (**a,b**) Number of collected cells in each organ for RNA (**a**) and ATAC (**b**) data. (**c,d**) Cell type proportions in each organ for RNA (**c**) and ATAC (**d**) data. The cell types were categorized into major groups for better visualization. (**e,f**) Donor gender proportions for RNA (**e**) and ATAC (**f**) data. (**g,h**) Donor age proportions for RNA (**g**) and ATAC (**h**) data. (**i,j**) Proportion of cells sequenced by different technologies for RNA (**i**) and ATAC (**j**) data.

In this paper, we presented hECA v2.0, an AI-ready ensemble cell atlas of scRNA-seq and scATAC-seq data on the basis of hECA v1.0. We expanded the hECA v1.0 atlas by incorporating 10 times more single-cell transcriptomics datasets, added a new modality—scATAC-seq, and systematically organized and structured the processed data to enhance usability for AI models. hECA v2.0 comprises 10,831,024 annotated human cells profiled by scRNA-seq from 90 published studies, and 1,450,511 cells profiled by scATAC-seq from 10 published studies, covering 40 and 25 organs, respectively^26–125^ (Fig. 1a,b). All data underwent uniform processing, and cell types were re-annotated using uHAF, resulting in 269 cell types for the transcriptomics data and 101 cell types for the chromatin accessibility data (Fig. 1c,d). The cells included in hECA v2.0 are distributed across all age groups and exhibit a balanced representation of donor gender (Fig. 1e–h). The data come from multiple sequencing platforms and are predominantly sequenced by 10X (RNA) and Combinatorial barcoding (ATAC) technologies (Fig. 1i,j). Compared to other representative single-cell data repositories, hECA v2.0 features in its comprehensive data harmonization and curation pipelines, which set it apart from mere data aggregation, and also features in its support for both transcriptome and epigenome data (Table 1).

**Table 1:** Comparison between hECA v2.0 and other representative single-cell data repositories.

	hECA v2.0	hECA v1.0^9^	DISCO v2^11^	HuBMAP^5^	TISCH2^18^	HCA data portal^3^	SCP^147^
Number of cells	12.3 million	1.1 million	135 million	Unspecified	6.3 million	64.4 million	63.2 million
Number of organs	42	38	144	73	20	209	104
Omics	RNA and ATAC	RNA	RNA	RNA, ATAC, etc.	RNA	RNA, ATAC, etc.	RNA, ATAC, etc.
Uniform processing of gene expression profiles	✓	✓	✓	✓	✓		
Cell-level metadata harmonization	✓	✓	✓				
Cell type harmonization	✓	✓	✓	✓	✓		
Manual curation	✓	✓	✓	✓	✓		
Cell-centric assembly	✓	✓	✓				

hECA v2.0 is designed to be an AI-ready single-cell data corpus. It served as the training dataset for the development of scMulan^126,127^, a generative pre-trained model that learns the cell language and demonstrates broad capabilities in generating, annotating, and reasoning over single-cell transcriptomes. The success of scMulan highlighted the value of hECA v2.0 as a robust foundation for AI-driven single-cell research. Designed with AI applications in mind, hECA v2.0 offers rich and consistently standardized metadata, which enable models to capture complex gene-attribute associations. Extensive manual curation and re-annotation using uHAF ensure label consistency across datasets and enhance biological interpretability. These characteristics position hECA v2.0 not only as a high-quality reference atlas, but also as a semantically structured and scalable resource optimized for training large-scale AI models and enabling *in silico* biological discovery.

## Methods

### scRNA-seq data collection and processing

#### scRNA-seq data collection

For the scRNA-seq data in hECA v2.0, we first searched for related articles using the keywords “single cell” plus a specific organ name, with the DOI number as the unique identifier. Only articles that generated new scRNA-seq data from human tissues were included.

For each selected article, we manually retrieved download links for the corresponding scRNA-seq data matrices. Datasets that were inaccessible due to access restrictions or lack of maintenance were excluded. Ultimately, we collected data from 90 studies, some of which contained multiple datasets, resulting in a total of 142 newly acquired scRNA-seq datasets. Details of these datasets are provided in Table S1.

#### Standardized processing of gene expression profiles

We processed all downloaded gene expression profiles using a unified workflow. First, all profiles were converted into gene-by-cell matrices. To unify the feature space (genes) across different datasets, we standardized gene names across datasets following the same strategy as in hECA v1.0^9^. Specifically, we compiled a comprehensive list of human gene symbols from the HUGO Gene Nomenclature Committee (HGNC) (https://github.com/XuegongLab/hECA-v2.0/blob/main/reference_genes.xlsx), and mapped the gene symbols in each dataset to HGNC-approved symbols using an in-house toolkit named GeneSymbolUniform (https://github.com/XuegongLab/hECA-v2.0/tree/main/Pytoolkit_GeneSymbolUniform). In practice, this tool handles three cases: for genes that were already represented in the standard names, we keep the expression values from the original dataset; for genes that were represented by aliases or former names, we assign their expression values to the corresponding standard gene name column; for genes not present in the dataset, we fill in zeros.

Compared to hECA v1.0, we updated the reference gene symbols from 43,878 to 42,117 by removing genes that were not approved by the HGNC committee or lacked an Ensembl ID from the reference set. Moreover, we updated the previous R-version GeneSymbolUniform to Python-version, in which we incorporated sparse matrix operations and parallel computing. In our test on a demo dataset with 33 K cells, the Python version reduced runtime from 22.6 minutes to 2.9 minutes and memory usage from 44.7 GB to 7.8 GB, demonstrating significantly improved processing efficiency and reduced computational costs.

After unifying gene symbols, we normalized the count matrices using the classical log-normalization approach: feature counts for each cell were divided by the total counts for that cell, multiplied by 10,000, and transformed using the natural logarithm^128^. After processing, each dataset’s gene expression profile was uniformly transformed into a normalized gene-by-cell matrix containing 42,117 genes.

#### Standardized generation of cellular metadata for scRNA-seq data

We manually curated and standardized cell-level metadata of 15 key attributes, including: “cell_id”, “study_id”, “seq_tech”, “donor_id”, “donor_gender”, “donor_age”, “donor_status”, “original_name”, “organ”, “region”, “subregion”, “sample_status”, “treatment”, “ethnicity” and “cell_type” (Table 2).

**Table 2:** Description of the standardized cellular metadata for scRNA-seq data.

Fields	Descriptions	Annotation rules
cell_id	The unique identifier of the cell.	Found in the data.
study_id	The DOI number of the study where the cell originates.	Found in the article.
seq_tech	The sequencing technology.	Found in the article or data.
donor_id	The unique identifier of the donor where the cell originates.	Found in the data.
donor_gender	The gender of the donor where the cell originates.	Found in the article or data. “M” for males, and “F” for females.
donor_age	The age of the donor where the cell originates.	Found in the article or data. For embryonic cells, use the format “E + embryonic day” (e.g., “E10.5”). For postnatal cells, use “P + postnatal day” (e.g., “P56”). For other cases, specify the age in months or years (e.g., “6mo”, “21 yr”).
donor_status	The health status of the donor where the cell originates.	Found in the article or data. “Healthy” or a specific disease name.
original_name	The cell type label in the original dataset.	Found in the data.
organ	The organ where the cell originates.	Found in the article or data. Fill in the organ name (e.g., “Brain”, “Heart”).
region	A division of the anatomical structure in the uHAF hierarchy where the cell originates.	Found in the article or data. Filled according to the uHAF ontology tree.
subregion	A subdivision of the anatomical structure in the uHAF hierarchy where the cell originates.	Found in the article or data. Filled according to the uHAF ontology tree.
sample_status	The condition or status of the sample where the cell originates.	Found in the article or data. “Normal” or a specific disease name.
treatment	The medical treatment received by donors with disease conditions.	Found in the article or data. Fill in corresponding medical treatments if “donor_status” was not “Healthy”.
ethnicity	The ethnicity of the donor where the cell originates.	Found in the article or data (e.g., “Asian”, “Latino”).
cell_type	The cell type name after re-annotation.	Filled according to the uHAF ontology tree (see “Unified cell type annotation for scRNA-seq data” for details).

Except for “cell_type”, all metadata were directly extracted from the original articles, their supplementary materials or datasets. The “organ”, “region”, and “subregion” fields were strictly annotated based on the uHAF macroscopic ontology we previously established^9^. We filled in “Unclassified” if corresponding information was not found.

To standardize cell type annotations across all datasets, we re-annotated cells separately for each dataset using a unified workflow (see “Unified cell type annotation for scRNA-seq data” for details). Cell type labels were assigned following our previously defined uHAF ontology, which provides human-curated cell type classifications and their markers for each organ^9^. We then filled the “cell_type” field with the re-annotated cell type labels.

#### Unified cell type annotation for scRNA-seq data

We harmonized cell type labels across different datasets by manually re-processing the data and re-annotating each cell following a unified workflow. To evaluate potential batch effects, we first normalized each dataset and performed dimensionality reduction to obtain Uniform Manifold Approximation and Projection (UMAP) embeddings. We then examined whether cells from different donors mixed together or showed clear separation by UMAP visualization. If donor-specific clustering was evident, we annotated the dataset at the donor level; otherwise, annotation was performed at the dataset level. In practice, to ensure annotation accuracy and avoid artificial biases, we conservatively split and annotated most datasets by donor.

Next, we performed quality control (QC), identification of highly variable genes, dimensionality reduction, clustering, and differentially expressed gene (marker gene) analysis for each split dataset. We referred to the cell types and marker genes defined in uHAF as the primary reference framework, and assigned cell type labels to the cell clusters accordingly. This ensured that the annotation was consistently guided. We expanded the uHAF ontology in hECA v2.0 by incorporating newly curated cell types and marker genes. This part of data processing was performed using the Seurat package in R^129^. Related data processing codes are available at https://zenodo.org/records/15638912.

#### Integration of scRNA-seq data across all datasets

We used the Python package scVI to integrate the scRNA-seq data across different datasets for visualization. Following the official tutorial, we used raw count matrices from all available datasets as input. To better capture cellular heterogeneity, we first selected 3,000 highly variable genes using the “sc.pp.highly_variable_genes” function from the Scanpy package in Python^130^, with the parameter flavor as “seurat_v3” and the original study labels specified as batch information for the parameter “batch_key”. We then trained an scVI model with two hidden layers (n_layers = 2) and a latent dimension of 30 (n_latent = 30), using a batch size of 512.

### scATAC-seq data collection and processing

#### scATAC-seq data collection

In hECA v2.0, we added scATAC-seq data as a new modality into the atlas. We searched for relevant articles using the keywords “single-cell ATAC, human” and included only those generating new scATAC-seq data. Articles without accessible data files in fragments, BED, or matrix formats were excluded. Ultimately, we collected scATAC-seq datasets from 10 studies, two of which are atlas-scale datasets, covering 25 organs. Details of these datasets are provided in Table S2.

#### Standardized generation of cellular metadata for scATAC-seq data

The cellular metadata for scATAC-seq data includes 14 attributes (Table 3), most of which align with those used for scRNA-seq data.

**Table 3:** Description of the standardized cellular metadata for scATAC-seq data.

Fields	Descriptions	Annotation rules
cell_id	The unique identifier of the cell.	Found in the data.
study_id	The DOI number of the study where the cell originates.	Found in the article.
ref_genome	Reference genome of the original dataset where the cell belongs. All the data were mapped to hg38 after processing.	Found in the article or data (e.g., “hg19”, “hg38”).
seq_tech	The sequencing technology.	Found in the article or data.
donor_id	The unique identifier of the donor where the cell originates.	Found in the data.
donor_gender	The gender of the donor where the cell originates.	Found in the article or data. “M” for males, and “F” for females.
donor_age	The age of the donor where the cell originates.	Found in the article or data. For embryonic cells, use the format “E + embryonic day” (e.g., “E10.5”). For postnatal cells, use “P + postnatal day” (e.g., “P56”). For other cases, specify the age in months or years (e.g., “6mo”, “21 yr”).
organ	The organ where the cell originates.	Found in the article or data. Fill in the organ name (e.g., “Brain”, “Heart”).
region	A division of the anatomical structure in the uHAF hierarchy where the cell originates.	Found in the article or data. Filled according to the uHAF ontology tree.
subregion	A subdivision of the anatomical structure in the uHAF hierarchy where the cell originates.	Found in the article or data. Filled according to the uHAF ontology tree.
sample_status	The condition or status of the sample where the cell originates.	Found in the article or data. “Healthy” or a specific disease name.
original_name	The cell type label in the original dataset.	Found in the data.
cell_type	The cell type name after re-annotation.	Filled according to the uHAF ontology tree (see “Unified cell type annotation for scATAC-seq data” for details).
markers	Marker genes used to re-annotate the cell.	Generated during re-annotation.

All metadata fields, except for “cell_type” and “markers”, were extracted from the corresponding articles, supplementary materials, and datasets. Similar to scRNA-seq data, we manually re-annotated cell types for scATAC-seq data to enhance cross-dataset consistency (see “Unified cell type annotation for scATAC-seq data” for details). The “cell_type” and “markers” fields were filled based on the re-annotation results. We filled in “Unclassified” if corresponding information was not found.

The uHAF ontology was shared between scATAC-seq and scRNA-seq data, ensuring a unified cell type classification system across modalities. A detailed description of the cellular metadata for scATAC-seq data is provided in Table 3.

#### Unified cell type annotation for scATAC-seq data

To harmonize cell type labels across different scATAC-seq datasets, we re-processed and re-annotated cells following a unified workflow. Given the varying data formats among datasets, we implemented two annotation workflows: one based on peak files and the other on fragment files. If a dataset was split by samples or donors, annotation was performed at the sample or donor level; otherwise, annotation was conducted at the dataset level.

We performed basic pre-processing of scATAC-seq data using the EpiScanpy package in Python^131^, which involved QC, feature selection (identifying highly variable peaks), log-normalization, dimensionality reduction (using Principal Component Analysis) and clustering (using the Leiden algorithm). We then constructed a gene activity matrix (GAM)—a gene-by-cell matrix—by summing the total counts of accessible peaks located within each gene’s 5 kb upstream region using EpiScanpy. The GAM was subsequently used to identify marker genes for each cell cluster.

We assigned cell types to each cluster based on uHAF ontology, following these principles:

1. Marker genes with smaller *p*-values were prioritized in cell type assignment.
2. A higher number of marker genes associated with a cluster increased the confidence of annotation.

To capture finer cell subtypes that might be missed in a single round of clustering, we performed a two-step clustering approach when necessary. Specifically, we first identified coarse-grained cell clusters, followed by a second round of dimensionality reduction, clustering, and differential expression analysis to refine subtypes, such as distinguishing CD8 + T cells from CD4 + T cells. Related data processing codes are available at https://zenodo.org/records/15638912.

#### Standardization of the chromatin accessibility profiles

To standardize the feature space across different scATAC-seq datasets for cell-centric assembly, we mapped all dataset-specific peaks in hECA v2.0 to our previously established consensus peaks (cPeaks)^132^. The cPeak set is a chromatin accessibility region reference constructed from 624 bulk ATAC-seq datasets, complemented by a set of putative accessible peaks predicted using a deep convolutional neural network. It contains 1.7 million accessible genomic regions with a median length of 525 bp. By projecting the heterogeneous and dataset-specific peaks onto this unified reference, we eliminated inconsistencies arising from peak calling in individual datasets and established a consistent cell-by-cPeak matrix. This transformation provides a standardized feature space that allows reliable comparison, integration, and downstream analysis across diverse scATAC-seq datasets.

For datasets in version GRCh38 (hg38), we converted the original peak features into overlapped cPeak features, defining the read counts of cPeak as those of overlapped original peaks. If a cPeak overlaps with more than one peak, the read counts from all overlapped peaks are added to the corresponding cPeak. This transformation converted the original cell-by-peak matrix into a cell-by-cPeak matrix. For the GRCh37 (hg19) datasets, we followed the same transformation process using the corresponding cPeak set in hg19 version. We then mapped the cell-by-cPeak matrix to hg38, so that the feature matrices of all datasets were in version hg38. We specified the hg19/hg38 version of the original datasets using the “ref_genome” key. This conversion resulted in a unified feature space comprising 1,657,194 genomic regions (https://github.com/XuegongLab/hECA-v2.0/blob/main/reference_peaks.zip).

#### Integration of scATAC-seq data across all datasets

We integrated scATAC-seq data across all datasets for visualization. We first filtered cPeaks using the “filter_genes” function from the Scanpy library. We retained cPeaks that are open in at least 0.5% of cells. We then used the “highly_variable_genes” method implemented in the Seurat package^129^ to select top 500,000 highly variable cPeaks. Subsequently, we employed the PeakVI model provided by “scvi-tools”. The batch information “study_id” is specified through the “scvi.model.PEAKVI.setup_anndata” function. During training, we set the batch size to 64 and used GPU. After training, we obtained the latent representation of the cells using the trained model. We then used the “sc.pp.neighbors” function to compute the cell-to-cell adjacency matrix based on the latent representations. Next, we computed the Uniform Manifold Approximation and Projection (UMAP) using the “sc.tl.umap” function, setting “min_dist” to 0.5 and “spread” to 1.5.

## Data Records

The complete, per-organ and per-project scRNA-seq and scATAC-seq data in.h5ad format have all been deposited in Zenodo^133–141^. A copy of all data has also been archived in OMIX, China National Center for Bioinformation / Beijing Institute of Genomics, Chinese Academy of Sciences (https://ngdc.cncb.ac.cn/omix) under accession numbers OMIX010495^142^ (scRNA-seq data) and OMIX010494^143^ (scATAC-seq data).

## Technical Validation

### Quality control of gene expression and chromatin accessibility profiles

We first assessed the quality of the processed transcriptomic and chromatin accessibility matrices. As many low-quality cells had already been filtered out in the original studies, the datasets we collected were inherently of relatively high quality. Nevertheless, we conducted standardized quality control (QC) procedures to ensure consistency. For the scRNA-seq data, we removed cells with either too few or too many detected genes, as well as those with a high proportion of mitochondrial gene expression. For the scATAC-seq data, we excluded cells with insufficient numbers of accessible peaks and discarded peaks that were accessible in too few cells. Dataset-specific thresholds were determined manually based on the data distribution (Tables S3-4).

**Fig. 2:**
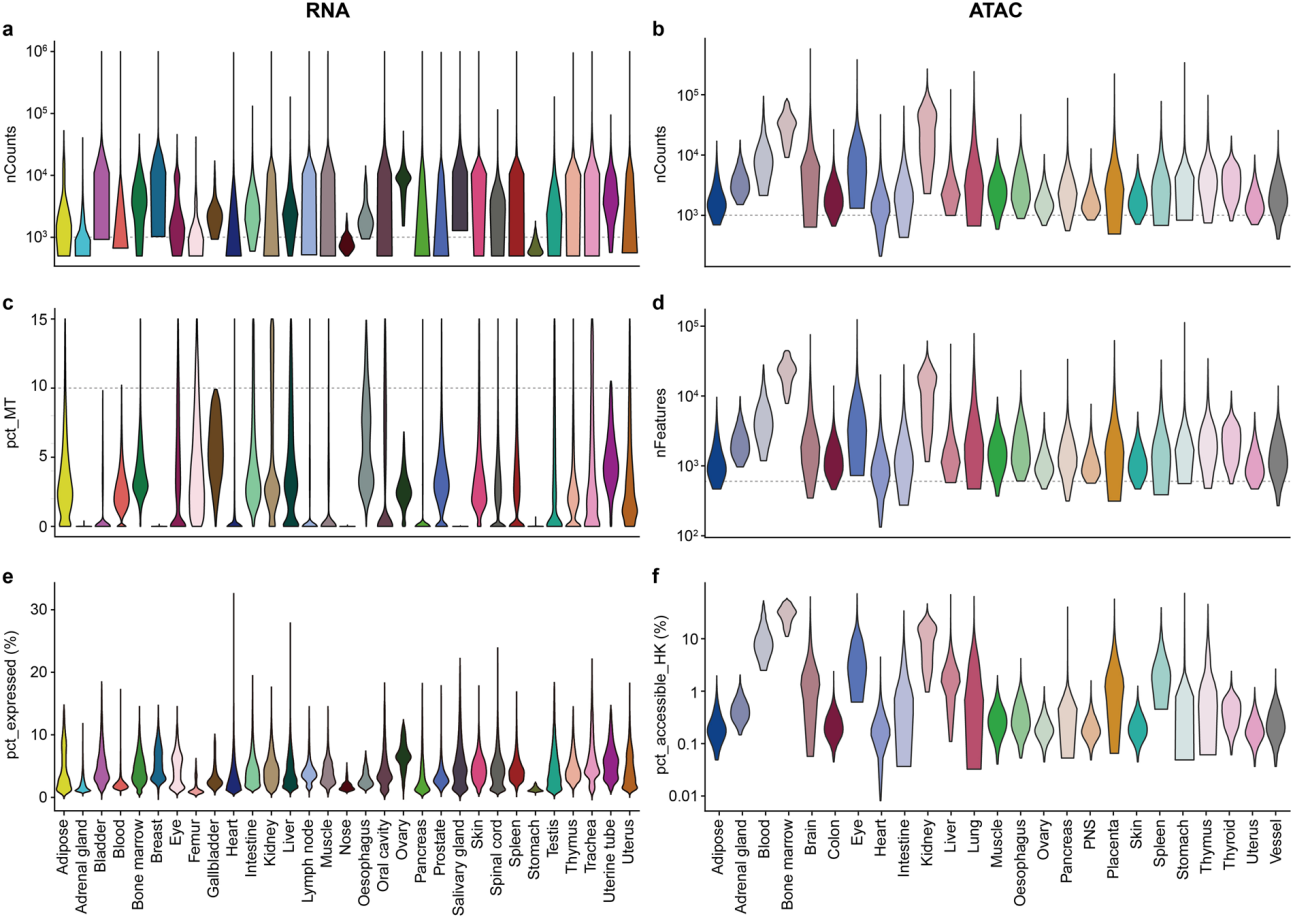
QC metrics for scRNA-seq and scATAC-seq data. (**a**) Total gene counts per cell in scRNA-seq data. (**b**) Total peak counts per cell in scATAC-seq data. (**c**) Percentage of mitochondrial gene counts (pct_MT) in scRNA-seq data. (**d**) Number of accessible cPeak features in scATAC-seq data. (**e**) Percentage of expressed genes (pct_expressed) per cell in the scRNA-seq data. (**f**) Percentage of accessible housekeeping genes (pct_accessible_HK) per cell in scATAC-seq data. All metrics were calculated using only cells with available count values. The dashed lines indicate the QC evaluation values described in the corresponding sections of the text.

After quality control, we evaluated the overall quality of the retained cells. Using cells with available count data, we found that 83.8% of cells in the scRNA-seq data have total counts larger than 1,000, and 92.6% have mitochondrial gene percentages below 10 (Fig. 2a,c). In the scATAC-seq data, we found that 97% of cells contain more than 1,000 peaks, and 99% have over 500 accessible peaks (Fig. 2b,d). These results confirm that the processed matrices are of high quality and suitable for downstream analyses.

To further validate the suitability of our data for AI model training, we assessed data sparsity and information content. Our analysis revealed that, on average, each cell expressed ~1,600 genes out of a total of 42,117 genes (3.8%) in the scRNA-seq data (Fig. 2e), and 3.6% of 24,538 housekeeping peaks were detected as accessible per cell in the scATAC-seq data (Fig. 2f). These values, considered in the context of a unified feature space that is substantially larger than that of any individual dataset, are consistent with the inherently sparse nature of single-cell omics. The results indicate that our data are representative and applicable in AI-based single-cell research.

### Validation of cell-level metadata

**Fig. 3:**
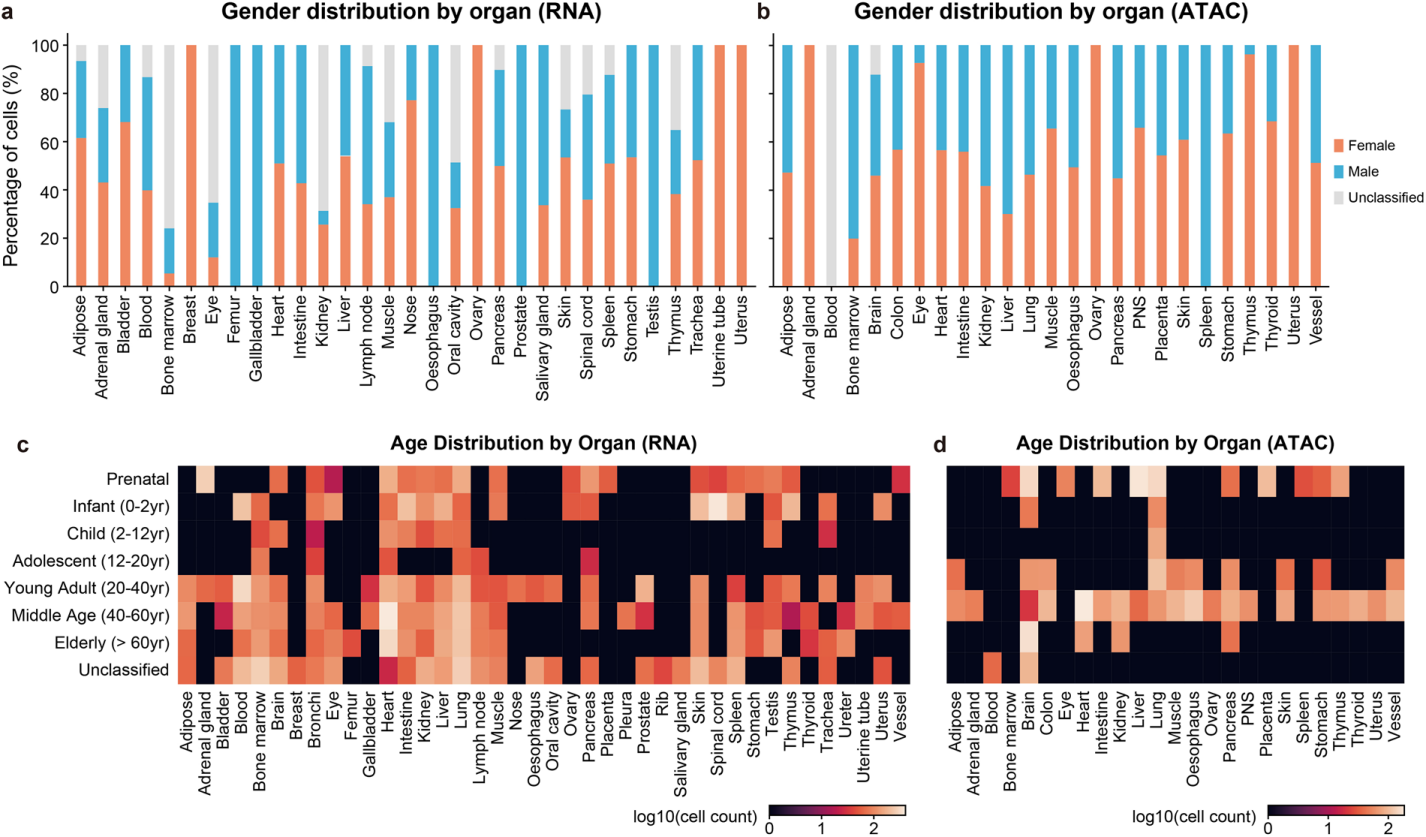
Evaluation of the cellular metadata for scRNA-seq and scATAC-seq data. (**a,b**) Gender distribution across organs in the scRNA-seq (**a**) and scATAC-seq (**b**) data. (**c,d**) Age distribution across organs in the scRNA-seq (**c**) and scATAC-seq (**d**) data. Values in the heatmap represent cell counts per organ per age group.

Another critical aspect for AI-ready data is the quality and consistency of cell-level metadata. We took substantial efforts to design and standardize the documentation of cellular metadata across studies (Methods). To rigorously assess metadata quality, we extracted and examined the values for each metadata field across all cells in both the scRNA-seq and scATAC-seq datasets. The results demonstrate that the metadata are well-structured and exhibit high consistency, making them directly usable as features or labels in downstream AI tasks (Fig. 1e–j, Fig. 3, Tables S5-6).

### Validation of cell type re-annotation

**Fig. 4:**
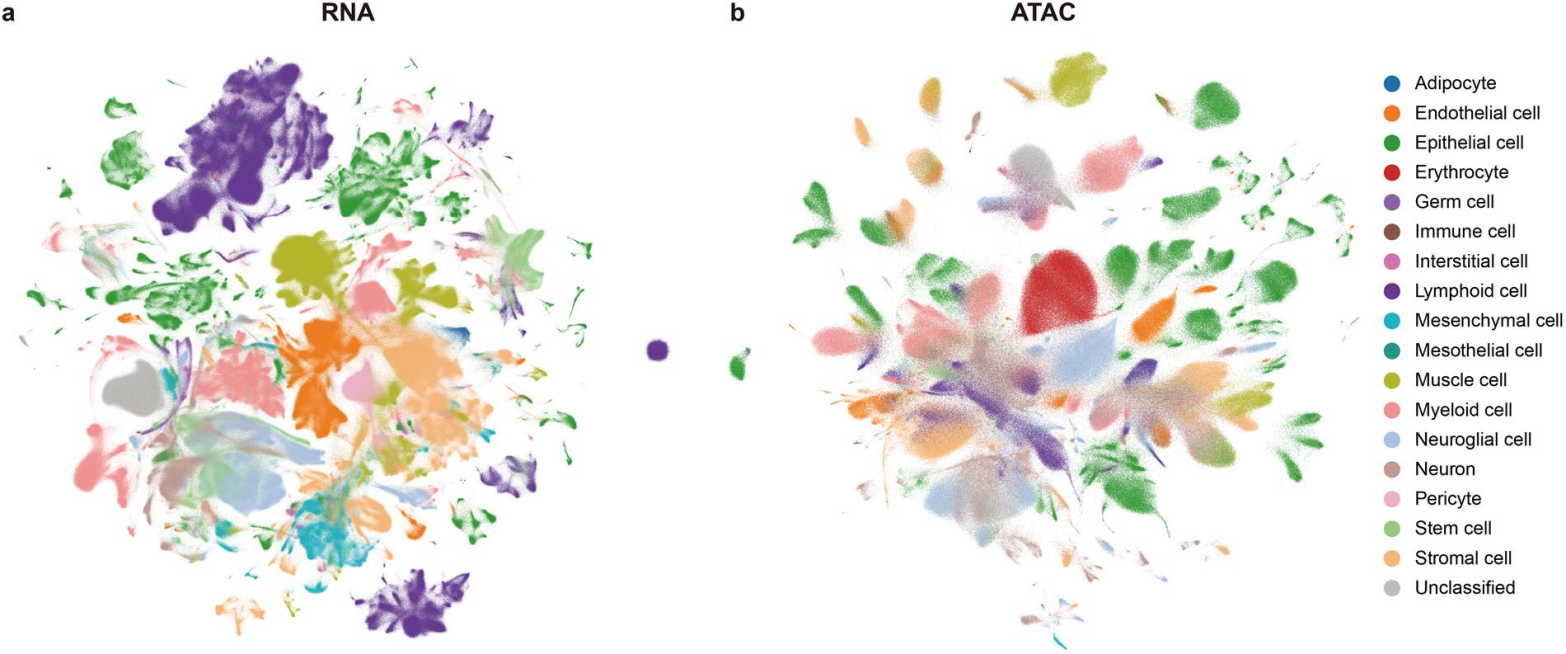
Visualization of cell type re-annotation for scRNA-seq and scATAC-seq data. (**a**) UMAP visualization of scRNA-seq data. (**b**) UMAP projection of scATAC-seq data. Cell types were aggregated into broader categories based on the uHAF hierarchy to improve clarity.

To ensure consistency of cell type labels across datasets, we developed a unified annotation framework (uHAF) and performed cell type re-annotation on a dataset-by-dataset basis (Methods). To validate the re-annotation results, we applied the scVI^144^ and PeakVI^145^ algorithms to integrate scRNA-seq and scATAC-seq data across different datasets, respectively, and projected cells into a low-dimensional space using Uniform Manifold Approximation and Projection (UMAP) (Methods). In both modalities, cells annotated with the same cell type predominantly cluster together, indicating high annotation consistency across datasets (Fig. 4a,b, Figs. S1–2).

## Usage Notes

The complete dataset, along with per-organ and per-project subsets, has been deposited and made publicly available on Zenodo. Users are encouraged to download the data files most relevant to their research needs. The hECA v2.0 data can support a wide range of applications, including but not limited to:

- Large-scale AI model training;
- Integrative single-cell analysis across organs and conditions;
- Reference mapping for cell type annotation;
- Organ- or system-specific mechanistic studies;
- Development and evaluation of computational methods.

To facilitate more convenient and flexible use of the data, we provide example scripts for converting h5ad-formatted files into other common formats (such as MTX matrices and Seurat objects) (https://github.com/XuegongLab/hECA-v2.0/tree/main/Usage_demo/h5ad_conversions), as well as a practical example of batch correction across samples and platforms (https://github.com/XuegongLab/hECA-v2.0/tree/main/Usage_demo/batch-correction-demo.ipynb).

## Supplementary information

Supplementary Figures and Table Legends

Table S1

Table S2

Table S3

Table S4

Table S5

Table S6

## Data Availability

All the processed scRNA-seq and scATAC-seq data are available at Zenodo^133–141^. A copy of the data is available at OMIX database (https://ngdc.cncb.ac.cn/omix) under accession numbers OMIX010495^142^ and OMIX010494^143^.

## Code availability

The Python toolkit GeneSymbolUniform and usage examples are available at https://github.com/XuegongLab/hECA-v2.0. Data processing codes for scRNA-seq and scATAC-seq data are available at Zenodo^146^.
