# Supplementary material for: hECA v2.0: an AI-ready ensemble cell atlas of single-cell RNA and ATAC sequencing data
Source: Sci Data. 2025 Dec 15;13:110. doi: 10.1038/s41597-025-06426-2 (PMC12852668; doi:10.1038/s41597-025-06426-2)
Supplement: Supplementary file 1 — Supplementary Figures and Table Legends [file 41597_2025_6426_MOESM1_ESM.docx]

# **Supplementary Figures**


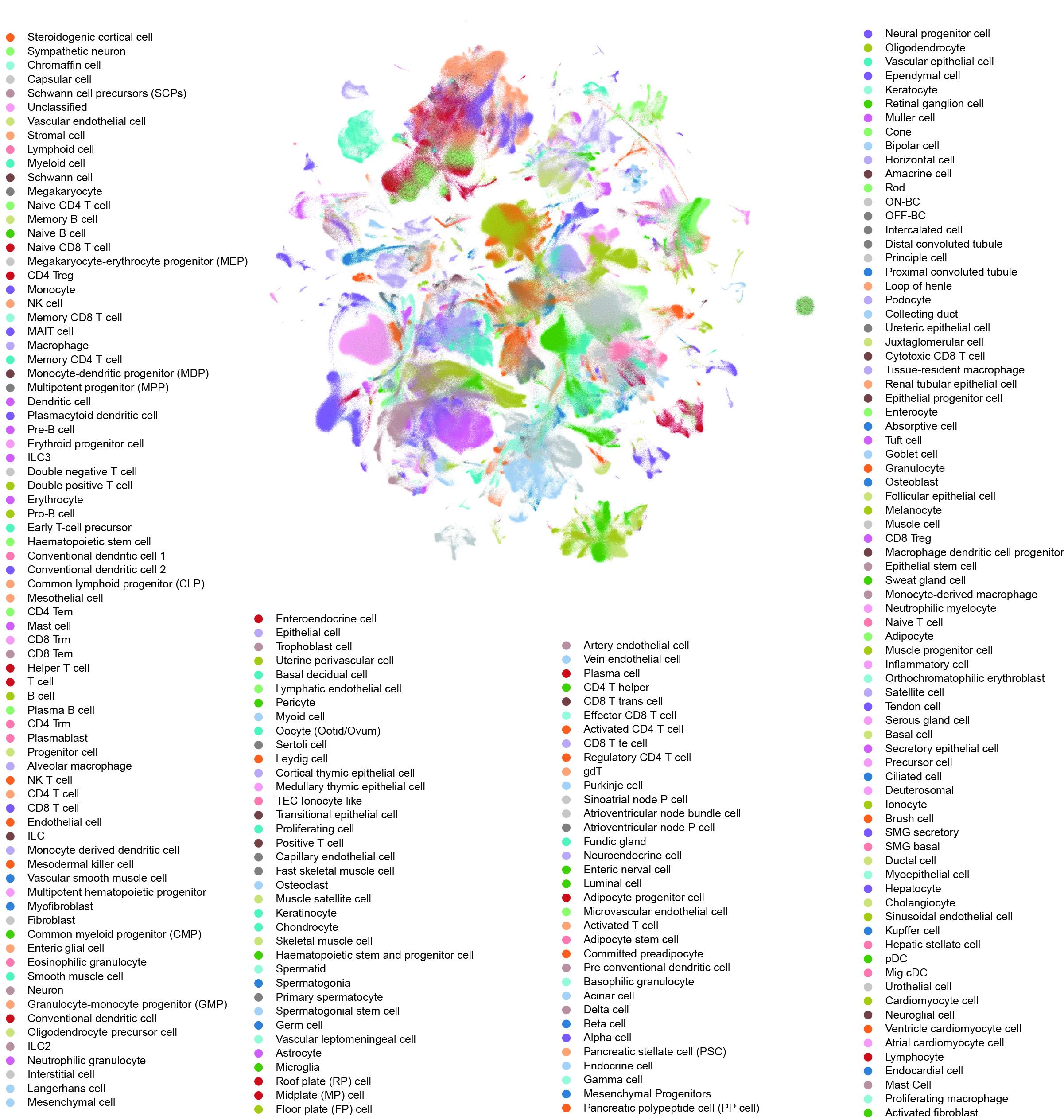


**Figure S1**. UMAP visualization of scRNA-seq data colored by cell types.


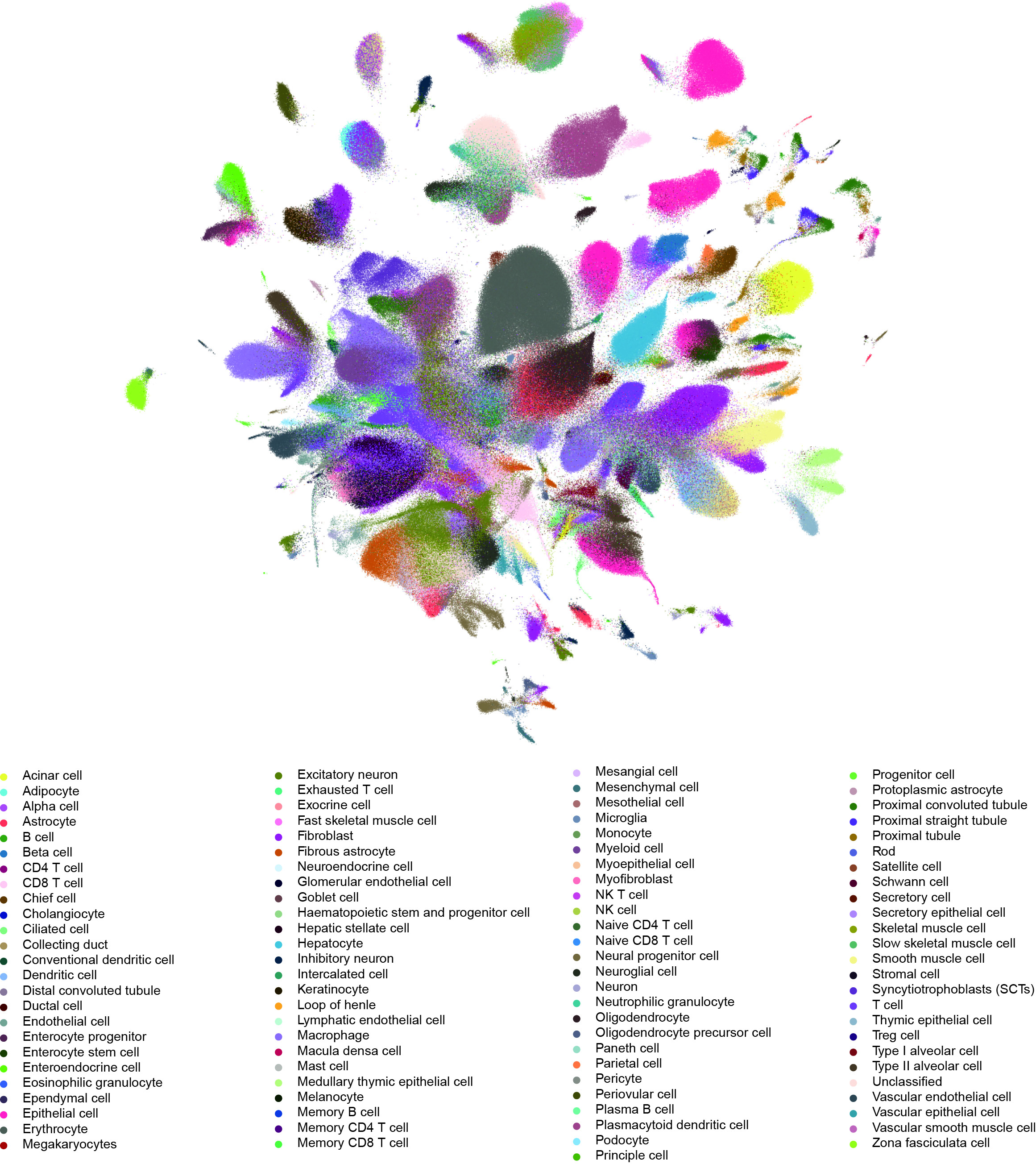


**Figure S2**. UMAP visualization of scATAC-seq data colored by cell types.

# **Supplementary Table Legends**

**Table S1.** Details of the collected scRNA-seq datasets in hECA v2.0

**Table S2.** Details of the collected scATAC-seq datasets in hECA v2.0

**Table S3.** Key parameters used in quality control and data processing for scRNA-seq data

**Table S4.** Key parameters used in quality control and data processing for scATAC-seq data

**Table S5.** Statistics of cellular metadata for scRNA-seq data

**Table S6.** Statistics of cellular metadata for scATAC-seq data
